# Prescribing of GLP-1 and GIP/GLP-1 Receptor Agonists and Other Glucose-Lowering Drugs Requiring Special Caution in Elderly Japanese People With Type 2 Diabetes: A Preliminary Report

**DOI:** 10.14740/jocmr6532

**Published:** 2026-05-31

**Authors:** Manaka Sato, Riku Takemura, Masahiro Yuki, Tomoya Saika, Michiko Yamazaki, Tsutomu Yamazaki, Masaya Sakamoto

**Affiliations:** a Department of Social Medical Sciences, Graduate School of Medicine, International University of Health and Welfare, Tokyo, Japan; b Department of Diabetes, Metabolism and Endocrinology, International University of Health and Welfare Mita Hospital, Tokyo, Japan; c Graduate School of Medical and Pharmaceutical Sciences, Chiba University, Chiba, Japan; d Department of Health and Social Behavior, School of Public Health, University of Tokyo, Tokyo, Japan; e Department of Hematology, Nippon Medical School, Tokyo, Japan

**Keywords:** Elderly, Type 2 diabetes, GLP-1RA, GIP/GLP-1RA, Prescription

## Abstract

**Background:**

Adverse drug events are more common in elderly people, prompting the Japan Geriatrics Society to update its “Guidelines for Safe Pharmacotherapy in the Elderly” in 2025. In this revision, glucagon-like peptide-1 receptor agonist (GLP-1RA) and glucose-dependent insulinotropic polypeptide/glucagon-like peptide-1 receptor agonist (GIP/GLP-1RA) were newly added to the list of drugs requiring special caution. This study aimed to descriptively investigate real-world prescribing patterns of GLP-1RAs and their concomitant use with other glucose-lowering drugs (GLDs) requiring special caution among elderly people with type 2 diabetes (T2D).

**Methods:**

This single-center, retrospective, cross-sectional, exploratory descriptive study included outpatients aged ≥ 65 years with T2D who received antihyperglycemic therapy between September and December 2025. Clinical characteristics, laboratory data, and prescription records were extracted from electronic medical records. Descriptive analyses were conducted stratified by GLP-1RA use (users vs. non-users) and by age categories (65 to < 75 years and ≥ 75 years).

**Results:**

Among 214 eligible people with T2D, 50 (23.4%) were prescribed GLP-1RAs. Among GLP-1RA users, 63.0% were classified as having obesity, and the mean number of concomitant GLDs was 3.2 ± 0.8. GLP-1RAs were often prescribed concomitantly with other GLDs requiring special caution in elderly people, including metformin, sodium-glucose cotransporter 2 inhibitor (SGLT2i), sulfonylureas, and insulin. In age-stratified analyses, oral semaglutide and dulaglutide were the most prescribed GLP-1RAs among people aged ≥ 75 years.

**Conclusions:**

GLP-1RAs were prescribed to approximately one quarter of elderly people with T2D and were most commonly used in people with obesity and more complex glucose-lowering regimens. Their frequent concomitant use with other drugs requiring special caution underscores the complexity of pharmacological management in elderly people.

## Introduction

Adverse drug events are known to occur more frequently and with greater severity in elderly people. In response to this concern, the Japan Geriatrics Society revised its “Guidelines for Safe Pharmacotherapy in the Elderly” [1] in July 2025, marking the first update in approximately a decade.

A key component of the revision was the update of the “List of Medications Requiring Special Caution in the Elderly.” This list focuses on older adults who are at particularly high risk of adverse drug events, including people aged ≥ 75 years as well as those aged < 75 years who are frail [2] or require long-term care. It was developed to prevent adverse drug events and to improve medication adherence through a reduction in medication burden. Among antidiabetic drugs, glucagon-like peptide-1 receptor agonist (GLP-1RA) and glucose-dependent insulinotropic polypeptide/glucagon-like peptide-1 receptor agonist (GIP/GLP-1RA) were newly added to this list. For the aims of this study, both glucagon-like peptide 1 (GLP-1) and GIP/GLP-1RA are collectively referred to as GLP-1RAs. The inclusion of GLP-1RAs in this list reflects growing concern regarding their safety profile in elderly people. Specifically, GLP-1RAs are associated with adverse events such as nausea, vomiting, diarrhea, biliary disorders, anorexia, and malnutrition, which may contribute to unintended weight loss [3, 4]. As a result, the guidelines emphasize the need to carefully assess the appropriateness of GLP-1RA therapy in elderly people, particularly those with frailty or sarcopenia, in whom excessive weight loss may lead to functional decline. When prescribing GLP-1RAs, clinicians are advised to monitor gastrointestinal symptoms, as well as unintended weight loss, particularly in people with frailty or sarcopenia [5, 6].

On the other hand, until the revision of the “Standards of Care in Diabetes 2022” by the American Diabetes Association (ADA), metformin was recommended as the sole preferred first-line medication [7]. As a result, the concomitant use of both agents has become common in real-world clinical practice in Japan. However, the Japan Diabetes Society’s “Recommendations for the Appropriate Use of Metformin” highlight that both metformin and GLP-1RAs are associated with gastrointestinal side effects, indicating that their combined use may require careful consideration. The concomitant use of these drugs may pose overlapping gastrointestinal safety risks, particularly in elderly people who are vulnerable to gastrointestinal symptoms. Nevertheless, because metformin is widely prescribed in routine clinical practice, concerns regarding gastrointestinal tolerability may not have led to changes in prescribing behavior, and concomitant use with GLP-1RAs may remain common. In addition, the guideline advises caution when prescribing metformin to people aged 75 years and older due to the risk of lactic acidosis [8].

Furthermore, the use of sodium-glucose cotransporter 2 inhibitor (SGLT2i) in the elderly is subject to caution. Specifically, these drugs should be prescribed carefully in people aged 75 years and older, or in those aged 65 to 74 years who are frail (e.g., with cognitive impairment, or reduced activities of daily living), due to the risks of dehydration and hypoglycemia [9].

Our study group previously investigated real-world prescribing patterns of sulfonylureas (SU), another class of glucose-lowering drugs requiring cautious use in the elderly [10]. That study revealed that high-dose SU prescriptions and the use of non-recommended agents remained common among elderly people, indicating that the risk of hypoglycemia is not yet adequately controlled in real-world settings. Building on these findings, the present study serves as a continuation of this line of research. Specifically, we aim to clarify the real-world prescribing patterns of GLP-1RAs, which were newly added to the list of drugs requiring special caution, and to examine their concomitant use with other glucose-lowering drugs that also require careful administration in the elderly, including SGLT2i, metformin, SU, and insulin.

This study aimed to investigate real-world prescribing patterns of GLP-1RAs descriptively and exploratorily, because they have been newly listed as drugs requiring cautions use in elderly people in Japan. Specifically, we also examined GLP-1RA prescribing and concomitant therapy patterns with other glucose-lowering drugs (GLDs) requiring caution, particularly among people aged ≥ 75 years. These findings may provide insights into the safe management of diabetes pharmacotherapy and serve as preliminary data for future multicenter longitudinal studies.

## Materials and Methods

### Study design and settings

This was a single-center, retrospective, cross-sectional exploratory descriptive observational study of elderly people aged 65 years and older with type 2 diabetes (T2D) who were receiving drug therapy and visited the outpatient of Tsuruoka Kyoritsu Hospital (Yamagata, Japan).

Clinical information from electric medical records, including vital signs, laboratory test results, and prescription records, was extracted from outpatient visits between September 1, 2025, and December 26, 2025, obtained from the outpatient clinics of three diabetologists.

Owing to the lack of prior studies directly aligned with the objectives of this research, no priori sample size calculation was conducted. This study was designed to generate descriptive real-world data to support hypothesis development and inform sample size determination in future multicenter longitudinal studies. Accordingly, all people who met the predefined inclusion criteria during the identification period were comprehensively included in the analysis.

In this study, the use of antihypertensive drugs, specifically calcium channel blockers, angiotensin II receptor blockers (ARBs), and β-blockers, as well as dyslipidemia drugs, including statins and fibrates, was confirmed. Each hypoglycemic, antihypertensive, and dyslipidemia drug was defined as “prescribed” if it had been prescribed for 14 consecutive days or more.

### Study population

**Figure 1:**
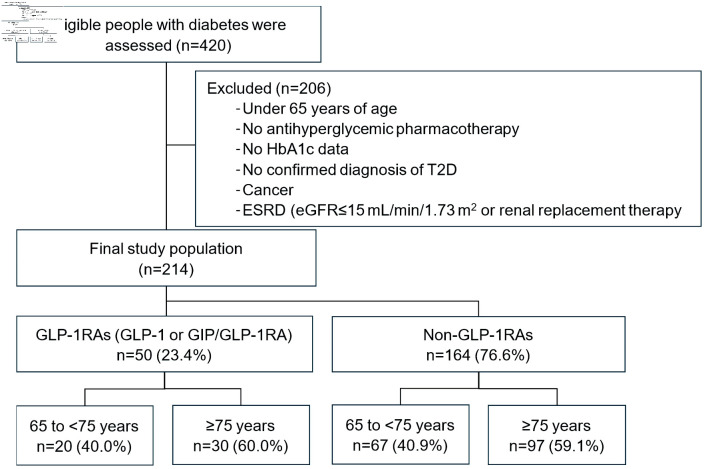
Flow diagram of patient selection and classification by GLP-1RA use. eGFR: estimated glomerular filtration rate; ESRD: end-stage renal disease; GIP/GLP-1RA: glucose-dependent insulinotropic polypeptide/glucagon-like peptide-1 receptor agonist; GLP-1RA: glucagon-like peptide-1 receptor agonist; T2D: type 2 diabetes; HbA1c: glycated hemoglobin A1c.

People were eligible if they had T2D, were ≥ 65 years of age, and were prescribed any antihyperglycemic drug, and had at least one available glycated hemoglobin A1c (HbA1c) measurement during the observation period. People were excluded if they had a diagnosis of type 1 diabetes, any cancer, or end-stage renal disease (ESRD), defined as an estimated glomerular filtration rate (eGFR) ≤ 15 mL/min/1.73m^2^ or undergoing renal replacement therapy (Fig. 1).

### Data analysis

Continuous variables are presented as mean with standard deviation (SD), and categorical variables as frequencies and percentages. Prescribing patterns were descriptively examined according to GLP-1RA use (users vs. non-users) and by age categories (65 to <75 years and ≥ 75 years). In this study, prescribing patterns refer to the frequency, types, and concomitant use of GLDs. As this study was exploratory descriptive study, a statistical significance test was not performed between the GLP-1RA and non-GLP-1RA groups, as well as between the 65 to <75 years and ≥ 75 years groups.

If people had multiple outpatient visits during the identification period, data from the most recent visit were used for data analysis. As this study aimed to provide a descriptive understanding of real-world practice patterns, no adjustments were made for potential confounders.

All data analyses were conducted using IBM SPSS version 31.

### Ethics approval statement

The protocol for this study was approved by the Ethics Committee of Tsuruoka Kyoritsu Hospital (number: 25024) on October 24, 2025 and conforms to the provisions of the Declaration of Helsinki and the Ethical Guidelines for Medical and Biological Research Involving Human Subjects issued by the Ministry of Education, Culture, Sports, Science and Technology (MEXT) and the Ministry of Health, Labor and Welfare (MHLW) of Japan, revised on April 1, 2024.

## Results

A total of 420 outpatients were assessed during the study period. After excluding people under 65 years of age, without antihyperglycemic pharmacotherapy, without HbA1c data, without a confirmed diagnosis of T2D, those with cancer, or those with ESRD, 214 people were included in the final analysis. Among them, 50 people (23.4%) were prescribed GLP-1RAs, while 164 people (76.6%) were treated with non-GLP-1RA therapies (Fig. 1).

### Baseline characteristics

The people included in the analysis had a mean age of 76.0 ± 6.5 years, and 59.3% were male. Overall, 40.7% were aged 65 to < 75 years and 59.3% were aged ≥ 75 years. The mean body mass index (BMI) was 24.9 ± 4.0 kg/m^2^, and people treated with GLP-1RA were more likely to have BMI ≥ 25.0 compared with non-GLP-1RA users (63.0%, 39.8%). HbA1c averaged 7.0±0.9%. High-density lipoprotein (HDL) cholesterol was lower in GLP-1RAs users than in non-users (52.7 ± 15.8, 58.2 ± 15.3 mg/dL). The mean number of GLDs was higher in GLP-1RAs users (3.2 ± 0.8, 1.9 ± 0.9).

Regarding the drugs of GLP-1RAs prescribed, oral semaglutide was used in 46.0% of cases, dulaglutide in 24.0%, injectable semaglutide in 18.0%, tirzepatide in 10.0%, and lixisenatide in 2.0%. Drugs associated with relatively less weight loss, such as oral semaglutide and dulaglutide [8, 9], were more frequently selected. With respect to concomitant drugs, GLP-1RA users were more frequently to be prescribed metformin (60.0%, 46.3%), SGLT2i (76.0%, 40.9%), SU (34.0%, 21.3%), and insulin (38.0%, 14.6%) compared with non-GLP-1RA users (Table 1).

**Table 1:** Characteristics of Elderly People With T2D, Stratified by Use of GLP-1RAs

	All (n = 214)		GLP-1RAs (n = 50)		Non-GLP-1RAs (n = 164)	
Age, years	214	76.0 ± 6.5	50	75.5 ± 6.5	164	76.2 ± 6.5
65 to < 75 years	87	(40.7)	20	(40.0)	67	(40.9)
≥ 75 years	127	(59.3)	30	(60.0)	97	(59.1)
Male	127	(59.3)	35	(16.3)	92	(43.0)
BMI, kg/m^2^	110	24.9 ± 4.0	27	25.6 ± 4.5	83	24.6 ± 3.8
< 25.0	60	(54.5)	10	(37.0)	50	(60.2)
≥ 25.0	50	(45.5)	17	(63.0)	33	(39.8)
HbA1c, %	214	7.0 ± 0.9	50	7.0 ± 0.7	164	7.0 ± 0.9
< 7.0%	109	(50.9)	24	(48.0)	85	(51.8)
7.0 to < 8.0%	86	(40.2)	23	(46.0)	63	(38.4)
≥ 8.0%	19	(8.9)	3	(6.0)	16	(9.8)
SBP, mm Hg	198	134.0 ± 16.0	46	132 ± 15.0	152	134.6 ± 16.3
DBP, mm Hg	197	74.7 ± 10.2	46	75.9 ± 8.7	151	74.3 ± 10.6
Total cholesterol, mg/dL	204	174.9 ± 28.3	47	169.1 ± 30.8	157	176.6 ± 27.3
Triglyceride, mg/dL	196	141.2 ± 79.3	46	137.2 ± 68.4	150	142.5 ± 82.5
HDL cholesterol, mg/dL	207	56.9 ± 15.5	49	52.7 ± 15.8	158	58.2 ± 15.3
LDL cholesterol, mg/dL	198	89.8 ± 25.5	46	88.0 ± 26.2	152	90.3 ± 15.8
eGFR, mL/min/1.73m^2^	202	66.5 ± 17.2	46	69.2 ± 17.6	156	65.7 ± 17.0
G1 + G2 (≥ 60)	27	(63.4)	31	(67.4)	97	(62.2)
G3a (45–59)	53	(26.2)	12	(26.1)	41	(26.3)
G3b (30–44)	19	(9.4)	3	(6.5)	16	(10.3)
G4 (15–29)	2	(1.0)	0	(0.0)	2	(1.3)
Number of GLDs	214	2.2 ± 1.1	50	3.2 ± 0.8	164	1.9 ± 0.9
GLP-1 and GIP/GLP-1RA	50	(23.4)	50	(23.4)	164	(76.6)
Oral semaglutide	23	(46.0)	23	(46.0)	–	–
Dulaglutide	12	(24.0)	12	(24.0)	–	–
Injectable semaglutide	9	(18.0)	9	(18.0)	–	–
Tirzepatide	5	(10.0)	5	(10.0)	–	–
Lixisenatide	1	(2.0)	1	(2.0)	–	–
Metformin	106	(49.5)	30	(60.0)	76	(46.3)
Metformin dosage, mg/day	106	809 ± 465	30	1,025 ± 539	76	724 ± 405
SGLT2i	105	(49.1)	38	(76.0)	67	(40.9)
SU	52	(24.3)	17	(34.0)	35	(21.3)
Insulin	43	(20.1)	19	(38.0)	24	(14.6)
DPP-4i	107	(50.0)	0	(0.0)	107	(65.2)
Antihypertensive drug	129	(60.3)	33	(66.0)	96	(58.5)
Dyslipidemia drug	131	(61.2)	25	(50.0)	106	(64.6)

Data are presented as mean ± standard deviation or n (%) referring to the population within each group. The numbers shown on the left side of each variable indicate the number of participants with available data for that variable; therefore, denominators may vary due to missing values. Percentages for BMI and eGFR were calculated using the number of people with available measurements as the denominator. T2D: type 2 diabetes; BMI: body mass index; SBP: systolic blood pressure; DBP: diastolic blood pressure; HDL: high-density lipoprotein; LDL: low-density lipoprotein; eGFR: estimated glomerular filtration rate; GLD: glucose-lowering drug; GLP-1RAs: glucagon-like peptide-1 receptor agonist and glucose-dependent insulinotropic polypeptide/glucagon-like peptide-1 receptor agonist; SGLT2i: sodium-glucose cotransporter 2 inhibitor; SU: sulfonylurea; DPP-4i: dipeptidyl peptidase-4 inhibitor; GIP/GLP-1RA: glucose-dependent insulinotropic polypeptide/glucagon-like peptide-1 receptor agonist.

### Age-stratified analysis

**Figure 2:**
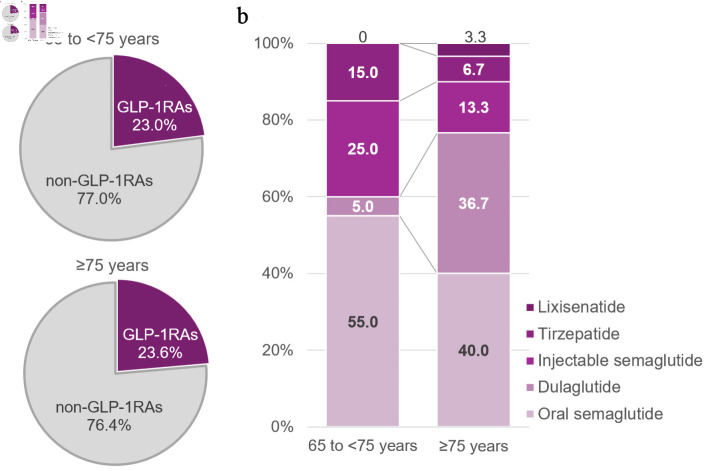
Prescribing frequency and type of GLP-1RAs by age group. (a) Percentages were calculated among all participants within each age group. (b) Percentages were calculated among GLP-1RA users within each age group. GLP-1RAs: glucagon-like peptide-1 receptor agonist and glucose-dependent insulinotropic polypeptide/glucagon-like peptide-1 receptor agonist.

**Figure 3:**
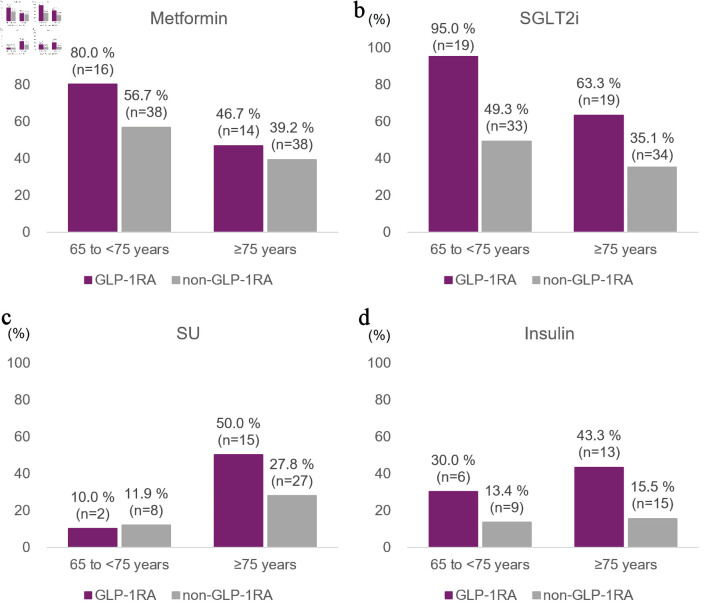
Concomitant use of glucose-lowering and cardiometabolic drugs by age group and GLP-1RAs treatment status. GLP-1RAs: glucagon-like peptide-1 receptor agonist and glucose-dependent insulinotropic polypeptide/glucagon-like peptide-1 receptor agonist; SGLT2i: sodium-glucose cotransporter 2 inhibitor; SU: sulfonylurea.

In people aged 65 to < 75 years (n = 87, 40.7%), those receiving GLP-1RAs had a higher mean BMI (27.1 ± 5.9, 25.3 ± 3.9 kg/m^2^) compared with non–GLP-1RA users. Glycemic control, as assessed by HbA1c, was similar between groups. GLP-1RA users (n = 20) received a greater number of GLDs compared with non-users (3.2 ± 0.7, 2.0 ± 1.1). Among this age group, GLP-1RA users were more frequently prescribed metformin (80.0%, 56.7%), SGLT2i (95.0%, 49.3%) and insulin (30.0%, 13.4%). Regarding the types of GLP-1RAs prescribed, oral semaglutide accounted for the largest proportion (55.0%), followed by injectable semaglutide (25.0%), tirzepatide (15.0%), and dulaglutide (5.0%) (Table 2, Figs. 2, 3).

**Table 2:** Age-Stratified Characteristics of GLP-1RAs users and Non-Users Among Elderly People With T2D

Variables	65 to < 75 years (n = 87)				≥ 75 years, (n = 127)			
	GLP-1RAs (n = 20)		Non-GLP-1RAs (n = 67)		GLP-1RAs (n = 30)		Non-GLP-1RAs (n = 97)	
Age, years	20	69.0 ± 3.1	67	70.1 ± 3.2	30	79.8 ± 4.0	97	80.4 ± 4.6
BMI, kg/m^2^	8	27.1 ± 5.9	38	25.3 ± 3.9	19	24.9 ± 3.8	45	24.0 ± 3.6
HbA1c, %	20	6.9 ± 0.6	67	6.8 ± 0.8	30	7.1 ± 0.7	97	7.2 ± 1.0
SBP, mm Hg	19	132.2 ± 13.7	65	131.4 ± 16	27	131.9 ± 16.1	87	137.0 ± 16.2
DBP, mm Hg	19	76.3 ± 8.9	64	76.3 ± 9.8	27	75.7 ± 8.7	87	72.8 ± 11.0
Total-cholesterol, mg/dL	18	175.1 ± 30.2	61	174.8 ± 26.9	29	165.4 ± 31.1	96	177.7 ± 27.7
Triglyceride, mg/dL	18	140.1 ± 76.4	62	157.6 ± 94	28	135.3 ± 64.2	88	131.8 ± 72.0
HDL cholesterol, mg/dL	19	52.7 ± 14.7	63	55.2 ± 14.4	30	52.7 ± 16.7	95	60.2 ± 15.6
LDL cholesterol, mg/dL	18	88.3 ± 29.2	62	88.0 ± 26.5	28	87.9 ± 24.7	90	91.9 ± 24.6
eGFR, mL/min/1.73 m^2^	19	67.7 ± 18.5	64	70.1 ± 17.1	27	70.3 ± 17.2	92	62.6 ± 16.3
Number of GLDs	20	3.2 ± 0.7	67	2.0 ± 1.1	30	3.2 ± 0.8	97	1.9 ± 0.9
Metformin dosage, mg/day	16	1,141 ± 619	38	796 ± 475	14	893 ± 413	38	651 ± 311

Data are presented as mean ± standard deviation. The numbers shown on the left side of each variable indicate the number of participants with available data for that variable; therefore, denominators may vary due to missing values. T2D: type 2 diabetes; BMI: body mass index; SBP: systolic blood pressure; DBP: diastolic blood pressure; HDL: high-density lipoprotein; LDL: low-density lipoprotein; eGFR: estimated glomerular filtration rate; CKD: chronic kidney disease; GLD: glucose-lowering drug; GLP-1RAs: glucagon-like peptide-1 receptor agonist and glucose-dependent insulinotropic polypeptide/glucagon-like peptide-1 receptor agonist; SGLT2i: sodium glucose co-transporter 2 inhibitor.

In people aged ≥ 75 years (n = 127, 59.3%), GLP-1RA users (n = 30) also received more GLDs compared with non-users (3.2 ± 0.8, 1.9 ± 0.9). Within this age group, oral semaglutide (40.0%) was most used, followed by dulaglutide (36.7%), injectable semaglutide (13.3%), tirzepatide (6.7%), and lixisenatide (3.3%). As shown in Figure 2, dulaglutide was prescribed with a frequency comparable to that of oral semaglutide among people aged ≥ 75 years, while other GLP-1RAs were used less frequently. GLP-1RA users were more frequently to be prescribed SGLT2i (63.3%, 35.1%), SU (50.0%, 27.8%), and insulin (43.3%, 15.5%) (Table 2, Figs. 2, 3).

## Discussion

This study investigated real-world prescribing patterns of GLP-1RAs among 214 elderly people with T2D at a single center, following their addition to the Japan Geriatrics Society’s “List of Medications Requiring Special Caution in the Elderly” [1]. Twenty-three percent of people received GLP-1RAs, and 59% of these users were aged 75 years or older. GLP-1RA users tended to have higher BMI and were prescribed a greater number of GLDs compared with non-users. GLP-1RAs were frequently used in combination with other GLDs that require cautious administration in the elderly, including SGLT2i, metformin, SU, and insulin, highlighting the complexity of pharmacological management in this population.

The results suggest that GLP-1RAs were selected for people with insufficient glycemic and weight control. GLP-1RA users had a higher prevalence of obesity and more frequent use of multiple GLDs, suggesting that clinicians introduced GLP-1RAs with expectations of glycemic improvement and weight reduction. In addition, a higher proportion of women was observed among GLP-1RA users compared with non-users. Previous systematic reviews and meta-analyses of randomized controlled trials have demonstrated that GLP-1RAs are associated with greater weight reduction in women than in men [11, 12]. Therefore, clinicians may have been more likely to prescribe GLP-1RAs to women in whom weight reduction was considered particularly beneficial. However, the underlying reasons for this sex-related difference could not be fully assessed in the present study. Notably, among people aged ≥ 75 years, dulaglutide was prescribed in approximately 40% of GLP-1RAs users, a proportion comparable to that of oral semaglutide (Fig. 2). The frequent selection of these drugs, which are generally associated with relatively modest weight reduction and related concerns such as frailty in the elderly, while still achieving glycemic control [13, 14], may reflect a prescribing preference for avoiding excessive weight loss and gastrointestinal symptoms in the elderly.

The higher use of SGLT2i among GLP-1RA users, along with lower HDL cholesterol and the use of antihypertensive and dyslipidemia drugs in 50–66% of people, suggests that clinicians may have considered future cardiovascular (CV) risk when selecting therapy. There were not many differences in blood pressure and lipid levels between GLP-1RAs users and non-GLP-1RA users. However, a review of drug history revealed that more than half of both groups had concomitant hypertension and/or dyslipidemia, suggesting that these complications were well-controlled with drug therapy in a high-CV-risk population. Both GLP-1RAs and SGLT2i have reported CV and renal protective effects [15], and their combination may have been chosen for elderly people with CV risk. In addition to their individual benefits, the complementary mechanisms of GLP-1RAs and SGLT2i, such as improvements in metabolic parameters, reductions in heart failure risk, and attenuation of renal progression, may offer additional benefits when used together, potentially enhancing overall cardiometabolic protection. However, SGLT2i carry risks such as dehydration, electrolyte imbalance, and renal function changes in the elderly [9], making careful monitoring of renal function and fluid status essential. The benefits and risks of combination therapy should be evaluated individually, balancing the potential for enhanced CV and renal protection with safety considerations in the elderly people.

Regarding metformin, the risks of lactic acidosis and gastrointestinal symptoms may require caution [8], especially in elderly people. In addition to these established clinical safety considerations, emerging experimental studies have suggested potential interactions between metformin and metal ions or amyloidogenic peptides; however, the clinical relevance of these findings to routine diabetes care remains uncertain [16, 17]. Because GLP-1RAs also carry a risk of gastrointestinal adverse events, concomitant use of metformin and GLP-1RAs warrants particular attention. In this study, metformin was used by about 40% of people aged ≥ 75 years, generally at relatively low doses, suggesting that clinicians were mindful of safety. Given the potential for overlapping adverse events, and the need for dose adjustments, individualized treatment strategies are essential when managing elderly people.

In this study, concomitant use of GLP-1RAs with SU or insulin was frequently observed even among people aged 75 years and older. Although these combinations were prescribed under the supervision of diabetes specialists with careful consideration of hypoglycemia risk, such regimens are generally regarded as requiring caution, particularly in the elderly, because their additive glucose-lowering effects may increase the likelihood of severe hypoglycemia [18, 19]. In elderly people, preventing hypoglycemia requires appropriate dose adjustment, self-monitoring of blood glucose, medication counseling, and regular follow-up.

This study has several important limitations that should be considered when interpreting the findings. First, this was a single-center study based on outpatient data from three diabetologists, therefore, the observed prescribing patterns may reflect local clinical practice and physician preference. As a result, the prevalence and combinations of GLP-1RA use reported in this study may not be fully representative of broader clinical settings, potentially limiting the external validity of the findings. Second, the sample size was limited, and detailed information on key patient characteristics, such as comorbidities and frailty status, was not comprehensively available. As a result, heterogeneity within this study population may not have been fully captured, and the observed prescribing patterns should be interpreted cautiously, recognizing that risk profiles and functional status differ across patients. Third, due to the cross-sectional design, causal relationships and long-term outcomes such as adverse events, CV events, and hypoglycemia could not be evaluated. Therefore, the results should be interpreted as a snapshot of prescribing patterns at a single time point, and no conclusions can be drawn regarding treatment trajectories or the clinical consequences of these prescribing decisions, including adverse events or long-term outcomes. Finally, this analysis did not assess GLP-1RA dosing, titration protocols, adverse events, or reasons for discontinuation, and thus provides limited insight into safety and dose optimization. This limits the interpretation of safety, tolerability, and clinical decision-making processes, and the findings primarily describe patterns of use rather than appropriateness or optimization of therapy. Future multicenter longitudinal studies are needed to validate these findings and evaluate safety in greater detail.

### Conclusions

This study descriptively characterized real-world prescribing pattern of GLP-1RAs among elderly people with T2D in a single clinical setting in Japan. GLP-1RAs were prescribed to approximately one quarter of the study population, most commonly in people with obesity and more complex glucose-lowering regimens. These were frequently used concomitantly with other GLDs requiring special caution in elderly people. Future multicenter longitudinal studies are needed to better contextualize these prescribing patterns.

## Data Availability

The datasets generated and analyzed during the current study are not publicly available due to institutional and ethical restrictions to protect patient confidentiality. The data supporting the findings of this study are available from the corresponding author upon reasonable request.

## Abbreviations

ADA: American Diabetes Association
ARB: angiotensin II receptor blocker
BMI: body mass index
CI: confidence interval
CV: cardiovascular
eGFR: estimated glomerular filtration rate
ESRD: end-stage renal disease
GIP/GLP-1RA: glucose-dependent insulinotropic polypeptide/glucagon-like peptide-1 receptor agonist
GLD: glucose-lowering drug
GLP-1RA: glucagon-like peptide-1 receptor agonist
HDL: high-density lipoprotein
LDL: low-density lipoprotein
MEXT: Ministry of Education, Culture, Sports, Science and Technology
MHLW: Ministry of Health, Labor and Welfare
OR: odds ratio
SBP: systolic blood pressure
SD: standard deviation
SGLT2i: sodium-glucose cotransporter 2 inhibitor
SU: sulfonylurea
T2D: type 2 diabetes
